# Mineral–Soil–Plant–Nutrient Synergisms of Enhanced Weathering for Agriculture: Short-Term Investigations Using Fast-Weathering Wollastonite Skarn

**DOI:** 10.3389/fpls.2022.929457

**Published:** 2022-07-22

**Authors:** Hiral Jariwala, Fatima Haque, Stephen Vanderburgt, Rafael M. Santos, Yi Wai Chiang

**Affiliations:** School of Engineering, University of Guelph, Guelph, ON, Canada

**Keywords:** enhanced rock weathering, isosilicate mineral, carbon-negative liming agent, soil carbon, negative emissions, calcimetry

## Abstract

Enhanced weathering is a proposed carbon dioxide removal (CDR) strategy to accelerate natural carbon sequestration in soils *via* the amendment of silicate rocks to agricultural soils. Among the suitable silicates (such as basalt and olivine), the fast-weathering mineral wollastonite (CaSiO_3_) stands out. Not only does the use of wollastonite lead to rapid pedogenic carbonate formation in soils, it can be readily detected for verification of carbon sequestration, but its weathering within weeks to months influences soil chemistry and plant growth within the same crop cycle of its application. This enables a variety of short-term experimental agronomic studies to be conducted to demonstrate in an accelerated manner what could take years to be observed with more abundant but slower weathering silicates. This study presents the results of three studies that were conducted to investigate three distinct aspects of wollastonite skarn weathering in soils in the context of both agricultural and horticultural plants. The first study investigated the effect of a wide range of wollastonite skarn dosages in soil (1.5–10 wt.%) on the growth of green beans. The second study provides insights on the role of silicon (Si) release during silicate weathering on plant growth (soybeans and lettuce). The third study investigated the effect of wollastonite skarn on the growth of spring rye when added to soil alongside a nitrogen-based coated fertilizer. The results of these three studies provide further evidence that amending soil with crushed silicate rocks leads to climate-smart farming, resulting in inorganic carbon sequestration, as well as better plant growth in agricultural (soybean and spring rye) and horticultural (green bean and lettuce) crops. They also demonstrate the value of working with wollastonite skarn as a fast-weathering silicate rock to accelerate our understanding of the mineral–soil–plant–nutrient synergism of enhanced weathering.

## Introduction

Overall global temperatures and the frequency of extreme weather events are rising due to an increase in atmospheric concentrations of carbon dioxide and other greenhouse gases (GHGs) (Fischer and Knutti, 2015). Thus, there is a pressing need to reduce GHG emissions, and carbon dioxide capture and storage (CCS) is seen as an essential strategy for reducing CO_2_ emissions as exemplified by the Mission Innovation initiative launched at the United Nations Climate Change Conference 2015 (Mission Innovation, 2015). Among several approaches to CCS, enhanced weathering is a proposed carbon dioxide removal (CDR) strategy to accelerate natural carbon sequestration in soils *via* the amendment of silicate rocks (Schuiling and Krijgsman, 2006). It is a chemical storage route whereby CO_2_ is converted into carbonates and bicarbonates by reaction with alkaline earth metal oxide-rich minerals (Lackner, 2003; Manning and Renforth, 2013; Khudhur et al., 2022). The most suitable class of naturally occurring Ca- and Mg-containing minerals for CCS are silicates, owing to the abundance, reactivity, and inertness of principal silicic by-product ([SiO_*x*_(OH)_4_-2*x*]*n*). There are several naturally occurring calcium and magnesium silicates suitable as mineral feedstock, for example, wollastonite (CaSiO_3_), enstatite (MgSiO_3_), olivine [a solid solution of forsterite (Mg_2_SiO_4_) and fayalite (FeSiO_4_)], diopside (MgCaSi_2_O_6_), and serpentine [(Mg,Fe)_3_Si_2_O_5_(OH)_4_] among other rocks such as basalt (Kwon et al., 2011; Paulo et al., 2021). Several independent research groups have recently reported on the increased inorganic carbon content of soils amended with alkaline minerals (Renforth et al., 2009; Renforth and Manning, 2011; Washbourne et al., 2015; Haque et al., 2019; Khalidy et al., 2021). Hence, using the alkaline mineral soil amendment to grow plants has the potential to combat anthropogenic emissions of CO_2_. Additionally, certain alkaline minerals have been shown to ameliorate soil quality and plant yield when added to soil, especially in nutrient deficient and highly weathered acidic soil (Mitani and Jian, 2005; Keller et al., 2012; Haynes, 2014; Meena et al., 2014).

The silicate rock used for this study contains two Ca-rich minerals: wollastonite (CaSiO_3_) in association with diopside (CaMgSi_2_O_6_). Among the wide variety of natural silicates suitable for the terrestrial weathering process, wollastonite is one of the most promising candidates because of its simple chemistry, high weathering rate, and ease of production of carbonated products, which is due to the weaker bonding of Ca ions to the silica matrix (Palandri and Kharaka, 2004; Schott et al., 2012). Wollastonite is widely distributed around the world, i.e., in China, Finland, India, Mexico, Spain, Canada, and the U.S., with a reserve size exceeding 100 million tons (Sangine, 2022). For wollastonite, the enhanced weathering route is explained in Equations 1–3. First, CO_2_ reacts with H_2_O to form a bicarbonate ion (${\text{HCO}}_{3}^{-}$) and a proton (H^+^) (Eq. 1). The metal ion (Ca^2+^) is liberated from the silicate by the proton (Eq. 2), and it ultimately reacts with the bicarbonate to precipitate as calcium carbonate (Eq. 3) (Hangx and Spiers, 2009). In soils, this process is referred to as pedogenic carbonate formation. Storage of carbonates in the near surface (i.e., topsoil) is expectedly temporary, subsequently migrating toward the subsoil, aquifers, and ultimately the final sinks of carbonates (e.g., ocean sediment) (Zamanian et al., 2016).


(1) $$\begin{array}{l} {\text{CO}}_{\text{2}}\text{ dissolution}:\;{\text{CO}}_{\text{2}(\text{g})}\text{ + }{\text{H}}_{\text{2}}{\text{O}}_{\left(\text{l}\right)}\;↔\;{\text{H}}_{\text{2}}{\text{CO}}_{\text{3}(\text{aq})}\;\\ \;↔\;{\text{HCO}}_{\text{3}}^{-}\text{ + }{\text{H}}^{\text{+}} \end{array}$$

(2) $$\begin{array}{l} \text{Calcium release from wollastonite}:\;{\text{CaSiO}}_{\text{3}(\text{s})}\text{ + }{\text{2H}}^{\text{+}}\;\to \\ {\text{ Ca}}^{\text{2+}}{\text{+H}}_{\text{2}}{\text{O}}_{\left(\text{l}\right)}\text{ + }{\text{SiO}}_{\text{2}(\text{s})} \end{array}$$

(3) $$\begin{array}{l} \text{Calcium carbonate precipitation}:\;{\text{Ca}}^{\text{2+}}\text{ + 2 }{\text{HCO}}_{3}^{-}\;\to \;\\ {\text{ CaCO}}_{\text{3}(\text{s})}↓\text{+ }{\text{H}}_{\text{2}}{\text{O}}_{\left(\text{l}\right)}\text{ + }{\text{CO}}_{\text{2}(\text{g})} \end{array}$$

Not only does the use of wollastonite lead to rapid calcium carbonate formation in soils, it can also be readily detected for verification of carbon sequestration, but its weathering within weeks to months influences soil chemistry and plant growth within the same crop cycle of its application. This enables a variety of short-term experimental agronomic studies to be conducted to demonstrate in an accelerated manner what could take years to be observed with more abundant but slower weathering silicates. This study presents the results of three complementary studies that were conducted to investigate distinct aspects of wollastonite skarn (ore of wollastonite containing secondary minerals including diopside) weathering in soils, in the context of both agricultural and horticultural plants, to accelerate our understanding of the mineral–soil–plant–nutrient synergism of enhanced weathering.

Soil fertility is an important factor in plant growth, and the primary nutrients required for plants are nitrogen (N), phosphorus (P), and potassium (K) (Havlin et al., 2017). Among primary nutrients, nitrogen (N) is an essential and crucial plant nutrient (Stewart et al., 2005). Nitrogen is a key element for the formation of proteins, chlorophyll, and other protein-carrying compounds in the plant. The deficiency of nitrogen in plants can cause a decrease in yield and crop development (Trenkel, 2010). For achieving targeted yield in crops, mainly, nitrogen-based fertilizers are applied in large quantities (Stewart et al., 2005). The widely used nitrogen fertilizers are urea, sodium nitrate, ammonium nitrate, potassium nitrate, and calcium ammonium nitrate (Nadarajan and Sukumaran, 2021). The use of traditional urea fertilizer causes the quick release of nutrients in the soil. The quick release of urea can cause severe damage to crops and the environment by nitrate loss in groundwater and ammonia volatilization (Azeem et al., 2014). Recently, growers are moving toward climate-smart agricultural practices and have started using coated/slow release/controlled release fertilizers for the nitrogen source (Jariwala et al., 2022). The plant also requires micronutrients, and among all micronutrients, silicon is considered a non-essential plant micronutrient (Epstein and Bloom, 2005). Moreover, the presence of silicon helps plants to increase yield and improve the thermal stability and tensile strength of natural fibers (Luyckx et al., 2017).

**Figure 1:**
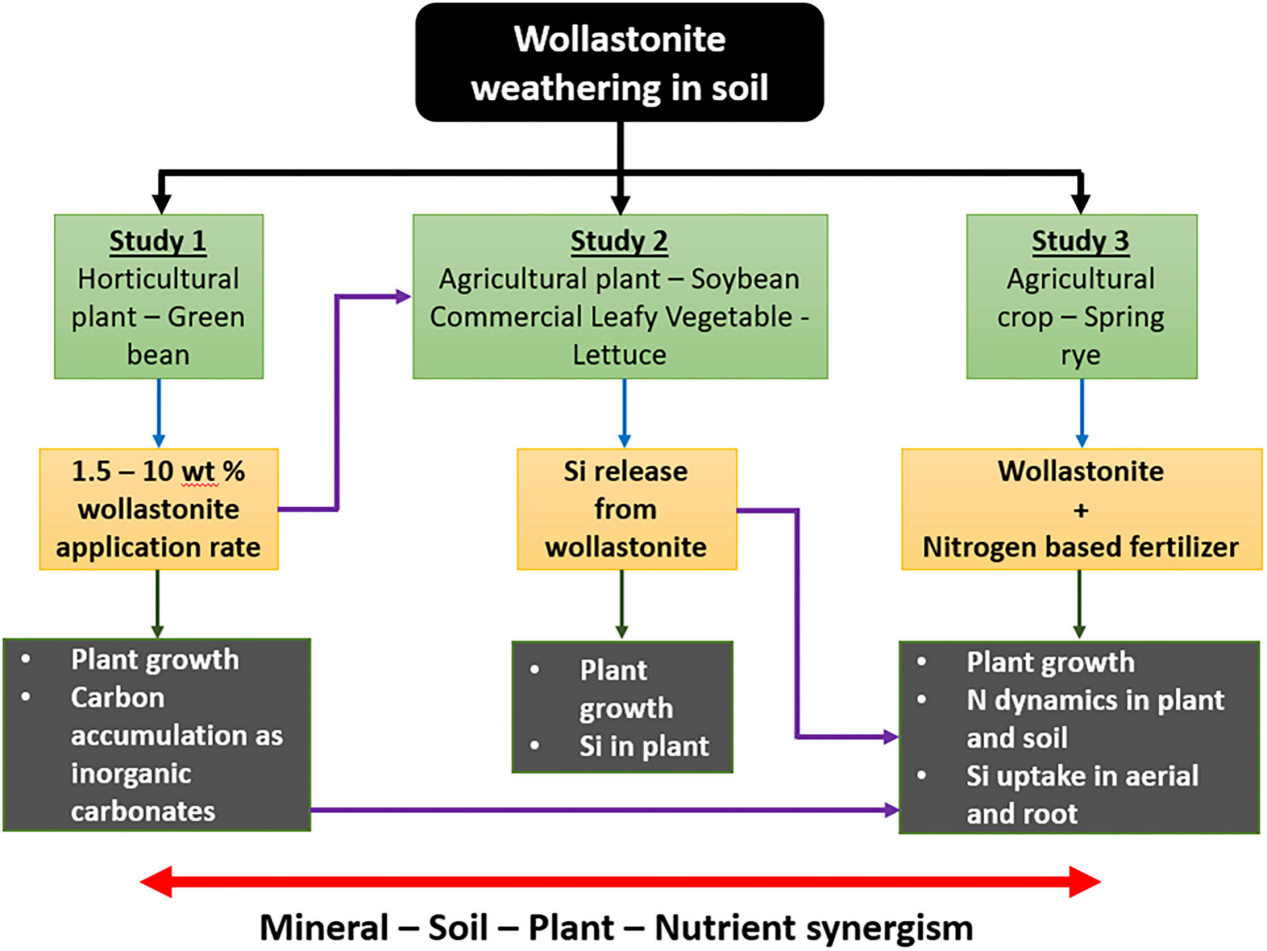
Schematic diagram of the flow of the current study. Study 1 investigated the synergism of wollastonite on plant growth and provided the optimal application rate for Study 2. Study 2 investigated the synergism of silicon release and uptake on improved produce quality. Study 3 further investigated wollastonite amendment and silicon uptake in synergism with nitrogen fertilizer. Study 3 also considered an alternate way of applying wollastonite to soil (as a fertilizer coating), achieving synergism between ease of application, fertilizer efficiency, and crop benefit.

The first objective of this study was to evaluate the benefit of mixing different dosages of wollastonite skarn as a soil amendment, the growth performance of green bean, a leguminous horticultural plant, and estimate carbon sequestration in the soil in terms of soil inorganic carbon (Study 1). The second study provides insights on the role of silicon (Si) release during wollastonite/diopside weathering on agricultural plant soybeans and a commercial plant, lettuce (Study 2). Soybean and lettuce were selected for study based on their economic importance in ontario agriculture and from personal communications with partner farmers who observed anecdotal benefits after using wollastonite in pilot projects (Haque et al., 2020a,b) that primarily studied carbon capture. The third study investigated the effect of wollastonite skarn on the growth of spring rye when added to soil alongside a nitrogen-based fertilizer. Rye (*Secale cereale*) is a non-leguminous cover crop and thus needs supplementation of a nitrogen-based fertilizer because it cannot fix atmospheric nitrogen on its own. The third study aimed to provide deeper insights into the dynamics of silicon uptake in plant biomass and nitrogen present in soil and plant biomass. The schematic given in Figure 1 illustrates the flow of the work.

## Materials and Methods

The wollastonite skarn used in this study was obtained from a quarry mine operated by Canadian Wollastonite in the village of Seeley's Bay (44°27′30″N, 76°15′20″W), located 30 km north of the city of Kingston (Ontario, Canada). In 2003, the ore reserve at this site was estimated to be 9 million tones at 41.3 wt.% wollastonite mean content (Grammatikopoulos et al., 2003). Skarn deposits are the most common source of wollastonite ore and are geologically formed through thermal metamorphic and metasomatic alterations of calcareous rocks, such as impure limestone (Berrada et al., 2011). The chemical, mineralogical, and morphological properties of milled wollastonite skarn used in this study have been reported in the study of Haque et al. (2020a) as follows. The main mineral phases, as determined using x-ray diffraction (XRD), were 49.0 wt.% wollastonite (CaSiO_3_) and 20.4 wt.% diopside (CaMgSi_2_O_6_), and the remainder included free SiO_2_ and minor silicates, aluminates, and sulfates; the calcite (CaCO_3_) concentration was 3.8 wt.%. The elemental composition, expressed as oxides and determined by wavelength dispersive x-ray fluorescence (WDXRF), was 52.5 wt.% SiO_2_, 29.8 wt.% CaO, 4.63 wt.% MgO, 4.04 wt.% Al_2_O_3_, 3.17 wt.% Fe_2_O_3_, 1.61 wt.% K_2_O, 1.57 wt.% Na_2_O, 1.30 wt.% SO_3_, 0.74 wt.% P_2_O_5_, 0.24 wt.% TiO_2_, and 0.19 wt.% SrO. The mean particle size of the milled wollastonite skarn, as determined by wet laser diffraction (Malvern Mastersizer SM), was 4.37 μm (calculated as the surface weighted (Sauter) mean diameter [D (3,2)], and 90% of particles by volume (D_90_) were <63.7 μm. The multipoint BET-specific surface area was determined to be 3.48 m^2^/g, as determined using a physisorption analyzer.

### Experimental Setup

For the first study, the experimental microplot was set up on the Thornbrough building rooftop at the University of Guelph, Ontario, Canada. Green bean plants were grown in wollastonite-amended soil (WAS) with four different wollastonite skarn doses (wollastonite in soil mass fractions), i.e., 1.5, 5, 7.5, and 10 wt.%. These dosages correspond, in areal terms (using a soil depth of 0.15 m and assuming soil bulk density of 1 ton/m^3^), to the range of 22.5–150 tones/hectare of wollastonite amendment. A control plot of unamended soil was also included in the experiment. The soil was collected from a commercial agricultural field (Woodstock, Ontario, Canada) and was classified as a sandy loam of orthic melanic brunisol origin, with a pH of 6.63, 3.2 wt.% of organic matter content, and an inorganic composition that included 65.06 wt.% SiO_2_, 3.03 wt.% K_2_O, and 2.02 wt.% P_2_O_5_ (as determined by wavelength dispersive X-ray fluorescence) (Haque et al., 2020a). A wide range of wollastonite skarn doses were tested on the experimental plots to determine an upper dosage that supports rapid carbon sequestration alongside healthy plant growth. The wollastonite powder was agitated in large buckets with the soil to achieve an even mixing of the amendment. Plots of the various WAS compositions (1.5–10 wt.%) without any plants were also maintained to check for wollastonite skarn weathering and carbonate accumulation under uncropped conditions and as such distinguish the effect of plants on the weathering processes vs. those of soil and ambient effects. Each microplot was 0.6 × 1.2 × 0.15 m and was filled with unamended soil or WAS. No other chemical, mineral, or organic amendments (e.g., solid or liquid fertilizers, limestone/dolomite, manure/compost, etc.) were used in these experiments. At the start of the experiment, the soil was supplied with adequate gardening water, and rainwater was the source of water during the rest of the experiment. The experiment was run for 14 weeks, between June and October. The maximum, minimum, and mean temperatures recorded during the experimental period were 25.7, 12.6, and 19.2°C, respectively.

The results of first study were used to choose the best application rate for wollastonite skarn (5 wt.%) to design the experiment for the second study. The optimal dosage was decided based on a combination of offering the best plant growth benefits while also achieving elevated levels of pedogenic carbonate formation. The main focus of this second study was to provide insights into the role of wollastonite skarn weathering on plant growth. This study was performed using potted plants at the Bovey Teaching Greenhouse (43°31′39.0″N 80°13′44.8″W) at the University of Guelph, where the average daily temperature was maintained at 18–20°C. The two plants chosen were soybeans and lettuce. A growth medium of sphagnum peat moss (Premier) was created by adjusting the pH to 6.0 using wollastonite powder. A control growth medium of peat moss adjusted to pH 6.0 with dolomitic limestone (CIL) was prepared for comparison. Both growth media contained ≈5 wt.% of either wollastonite skarn or dolomitic limestone. Ten pots each for WAS and control were maintained for both the plants. Plants were protected from insects by an enclosure and given adequate water daily. A commercial 18-18-21 fertilizer (Miracle-Gro) was given equally to all pots as per manufacturer instruction. The trial was run during the months of July and August and ended after 53 days.

For the third study, spring rye (*Secale cereale*) was used as the experimental plant and it was grown in wollastonite-amended soil along with a nitrogen-based fertilizer. Spring rye is a crop typically used as a cover crop (to protect the soil during the winter period) and is typically planted in late Fall and harvested in Spring. The aim of this study was to understand the effect of wollastonite skarn on plant growth when co-applied specifically with a nitrogen fertilizer. This is in contrast with the previous two studies where fertilizers were not added in the first study, and a composite fertilizer was used in the second study. Also, rather than applying powdered wollastonite by broadcasting, in this study, the wollastonite was coated onto the fertilizer pellets for a true co-application (more details later). The spring rye setup was in the greenhouse, and the temperature was 15–22°C from November to May. Growing pots were filled with unfertilized Garden Club™ topsoil (soil pH 5.47). Each pot was filled with 25 kg of topsoil, and 15–20 vol.% soil moisture was maintained throughout the experiment by watering two times a week. The seeds were received from Ontario Ministry of Agriculture, Food and Rural Affairs (OMAFRA). The seeding rate for each pot was 110 kg·ha^−1^, which corresponds to 1.75 g of seeds that were applied using the broadcasting method on 0.16 m^2^ of pot area equally (OMAFRA, 2021). Seed germination was observed after 9 days of sowing the seeds. The spring rye experiment was started in the first week of November, and germination was completed by the end of November. The plants were fully grown by subsequent May and harvested in the first week of June. A single application of fertilizer was performed in mid-December at a nitrogen rate of 57.5 kg·ha^−1^ (recommended rate is 55–80 kg·ha^−1^) with the broadcasting method (OMAFRA, 2021). The following treatments were made for the spring rye setup: (1) control (using topsoil and no application of fertilizer); (2) uncoated urea (no wollastonite); and (3) coated fertilizer (with wollastonite). Coated nitrogen fertilizer (urea (46-0-0) with organo-mineral coating) was prepared, and its efficacy was compared with uncoated urea. For uncoated treatment, 2.0 g of urea (46% N, 46-0-0) was applied per pot, and for the coated fertilizer treatment, 3.2 g of the developed fertilizer (62.85 wt.% urea, 18.85 wt.% wollastonite skarn, and 18.30 wt.% organic binder) was applied, resulting in equal nitrogen application (0.92 g N per pot).

### Analysis Methods

#### Plant Analyses

Plant growth across the three studies was analyzed based on the plant height, stem width, leaf blade width, root biomass, and aerial fresh and dry biomass. At the end of an experimental run, the plants were harvested by cutting them just above the soil level and were stored in paper bags at room temperature. The root biomass of each plant was determined by separating the roots from the soil by washing them with sufficient water. Air-dried plant samples were prepared by placing them in aluminum foil containers separately and dried at 80°C for 24 h in a BINDER-ED56 forced-air oven (Kalra et al., 1998). For green beans, soybeans, and spring rye, the pods/spikes were removed before weight measurement. The yield of the green bean pods, soybean pods, lettuce, and spring rye spikes was also determined in terms of dry weight.

For the second and third studies, analysis of silicon from plant samples (soybeans, lettuce, and spring rye) was done based on a digestion method using KOH (Cai et al., 2008; Frantz et al., 2008; Bossert et al., 2019). The plant samples were ground using a stainless-steel grinder until they became a fine powder. Two samples (aerial part and root) were ground separately. Ground samples were collected in a plastic tube, avoiding the use of glassware during the experiment to prevent silica contamination from a glass surface. A portion of each ground sample was weighted (100–120 mg) and transferred to PTFE (Teflon®) digestion tubes. A volume of 5 ml of 7.5 M KOH was added to initiate the first step of digestion, and eight tubes were placed inside a Titan MPS™ Microwave Sample Preparation System. During the first digestion, samples were digested at 200°C for 45 min. After the first digestion, PTFE tubes were cooled in a water bath for 1 h; then, for the second step of digestion, 2 ml of H_2_O_2_ (30%, Fisher) was added as an oxidizer, and tubes were again placed in the digester at 200°C for 30 min. After completing the digestion, extracted liquid for each sample was diluted using 0.1 M NaOH to meet the calibrated range of the AAS (Atomic Absorption Spectroscopy), between 100 and 500 ppb. For AAS analysis, the Shimadzu AA-6300 AAS was used along with Graphite Furnace Atomizer (GFA-EX7i) and autosampler ASC-6100. Silicon bulb wavelength was set at 251.611 nm (slit width-0.7 nm), low current of 20 mA was set to avoid overheating of the Si-lamp, and the sample size for all samples was set at 10 μl. The data we calculated using standards' calibration and the silicon content was expressed in %.

For the lettuce wilt test in the second study, one leaf of lettuce was harvested by cutting at the soil base from each pot of the control (*n* = 10) group and the wollastonite (*n* = 10) group. The leaves were immediately transferred to individual plastic trays, adaxial side up, and then placed into a refrigerator at 7°C and 65% humidity. The starting mass of each leaf was measured, with subsequent measurements every 12 h for 4 days. Measurements were continued every 24 h for another 9 days, after which moisture loss ceased.

For the third study, plant nitrogen content was determined. The spring rye plant nitrogen content was analyzed using a CHNS-O analyzer (Dhaliwal et al., 2014). All samples were analyzed using a Thermo Scientific™ CHNS/O Flash 2000 analyzer. The biomass samples were dried in the oven at 60°C for 48 h to remove moisture and later ground using a grinder.

#### Soil Analyses

The soil inorganic carbon content (calculated as CaCO_3_-equivalent) was determined by calcimetry, a volumetric method. The soil samples were suspended in Milli-Q water (5 g in 20 ml), to which 7 ml of 4 M HCl was added in a sealed Erlenmeyer flask connected to a graduated water-filled manometer column that recorded the volume of released CO_2_ (Eijkelkamp Calcimeter 08.53) (Chen et al., 2015). The net gain of CO_2_ accumulated in the soils (as CaCO_3_-equivalent) was determined as the difference between the values determined from calcimetry at the end of each experiment and the initial carbonate loading of each soil (including carbonates originally contained in the soils and in the amended wollastonite skarn).

Soil moisture was monitored using an Extech-MO750 Soil Moisture Meter. The pH of soils was determined by agitating soil with 0.01 M CaCl_2_ solution (1:5 mass ratio) for 30 min, settling for 60 min, and recording the pH of the supernatant (Pansu and Gautheyrou, 2007).

Soil ammonium and nitrate were extracted using the soil-water extraction method (Saha et al., 2018) and analyzed using a Cole-Parmer® Combination Ion-Selective Electrode (ISE) for ammonium (RK-27502-03) and nitrate (RK-27503-24). Total inorganic nitrogen (ammonium + nitrate) was calculated as the sum of the two independent analyses (Xu et al., 2019).

#### Statistical Analysis

For the microplot Study 1, each plot had *n* = 30 plants and there was one plot per treatment. For the soybean and lettuce in Study 2, each treatment had *n* = 10 plants. For the spring rye study, treatments were made in triplicate pots, and each pot had *n* = 30 plants. All plant and soil analysis readings were made in triplicate, and the mean results reported herein are represented along with their standard deviations. The data were statistically analyzed using paired *t*-test, a one-way analysis of variance (ANOVA) with Tukey's HSD (honestly significant difference) test, and Pearson's correlation coefficient test. A *p*-value < 0.05 was used as the threshold for statistical significance. Data analysis was performed using SPSS Statistics 26 (IBM) software and the statistical tool XLSTAT (Addinsoft). The data visualization was carried out using OriginPro 2021b (OriginLab) and R-4.0.5 (R Core Team).

## Results and Discussion

### Study 1—Effect of Wollastonite Skarn Soil Amendment Dosage on Plant Growth (Green Beans) and Pedogenic Carbonate Accumulation

**Figure 2:**
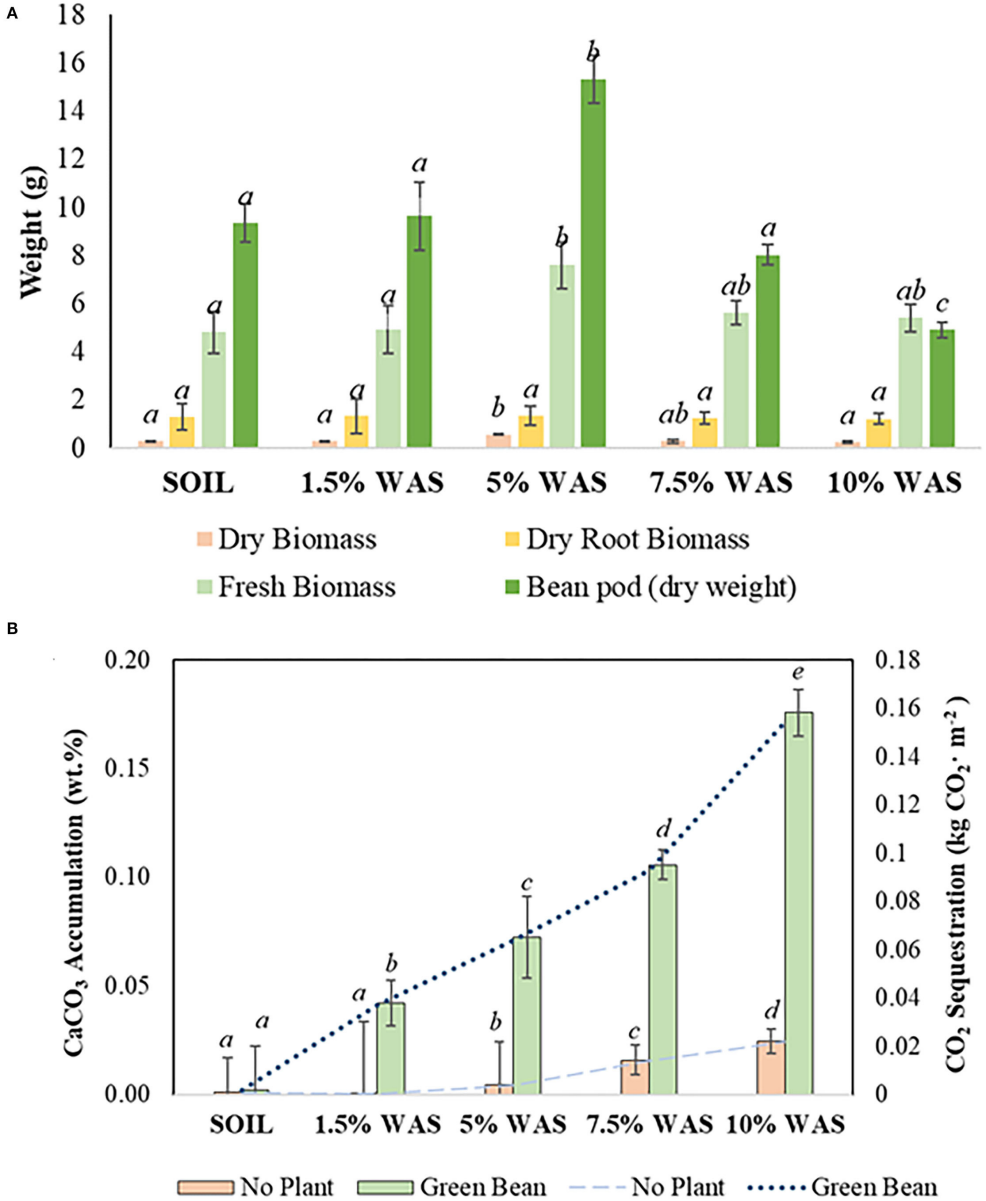
**(A)** Variation in the green bean fresh and dry biomass (*n* = 15), dry root biomass (*n* = 15), and pods (analyzed per subplot of five plants) at different doses (wt.% in soil) of wollastonite skarn soil amendment; **(B)** Pedogenic carbonate accumulation (bars) and CO_2_ sequestration (lines) in the various microplots. Lowercase letters (a–e) indicate significant differences between treatments (*p* < 0.05).

Figure 2A shows the variation in fresh and dry biomass, dry root biomass, and pod dry weight among green bean plants grown under different wollastonite skarn dosages using a sample size (*n*) of 15 plants. The green bean trials showed that the plants performed best in the 5 wt.% WAS, with the plants exhibiting increased plant biomass weight (+57.7% fresh and +88.5% dry) and root biomass (+4.1%) in comparison with those grown in the control plot; these differences were significant (*p* < 0.05). There was no significant change in the stem width (3.2 ± 0.09 mm), leaf blade width (25.0 ± 0.9 mm), or plant height (21.8 ± 4.2 cm), thus indicating that plants performed well at the various doses of wollastonite skarn. The green bean yield was highest in the 5 wt.% WAS (+64% vs. the control), while the yield was lower (−14%) in the 7.5 wt.% WAS, and the lowest yield was obtained in the 10 wt.% WAS (−47% vs. the control). At the end of the growth trial, the pH of the 5 wt.% WAS microplot was 7.16 ± 0.03, which is within the suitable range for green bean growth (pH 6.5–7.5) (Ketterings et al., 2005). The pH of the 7.5 wt.% WAS plot nearly reached the upper limit of the suitable pH range (7.45 ± 0.01), which helps explain the lower yield. In the 10 wt.% WAS microplot, the final pH was 7.58 ± 0.06 and thus exceeded the range at which bean plants effectively absorb essential nutrients from the soil.

Overall, the inorganic carbon content of the WAS, measured in terms of newly formed pedogenic calcium carbonate (CaCO_3_), was found to be higher than that in the control microplots. The CaCO_3_ amounts accumulated in the WAS are provided in Figure 2B. The accumulated amount of CaCO_3_ increased with increasing wollastonite skarn dosage. At the end of 14 weeks, the highest accumulation, +0.18 CaCO_3_ wt.%, was observed in the 10 wt.% WAS microplot; this value is equivalent to 1.5 tones CO_2_·ha^−1^ sequestration. In this study, the lowest CaCO_3_ accumulation in the planted microplots occurred in the 1.5 wt.% WAS microplot, and there was no significant difference in the CaCO_3_ content in the untreated soil (*p* > 0.05). This lack of accumulation of pedogenic carbonates can be tied to the lower pH of this WAS of 6.58 ± 0.16. This can prevent precipitation of carbonates but can allow for migration of bicarbonates to the soil leachate (not monitored); thus, weathering of wollastonite/diopside likely still occurred in this treatment. At the remaining WAS levels (5–10 wt.% WAS), the accumulated CaCO_3_ content was significantly higher (p <0.05) than that in the control soil. In the soils with no plants, the CO_2_ sequestration value did not surpass +0.03 CaCO_3_ wt.% in 10 wt.% WAS, confirming the significant role of plants in accelerating the weathering of wollastonite/diopside in soils (Haque et al., 2019). This occurs because, under cropped conditions, organic acids are produced from the plant roots; these acids facilitate the dissolution of wollastonite/diopside, hence increasing the release of calcium ions in the soil that further react with the dissolved CO_2_ present in the soil (as bicarbonates) to form calcium carbonate.

The results of this microplot experiment are significant to determine the potential for climate change mitigation *via* wollastonite skarn weathering in agricultural soils. This study demonstrated that, after 14 weeks of exposure to ambient atmospheric conditions in Ontario, pedogenic carbonate accumulated in the WAS (and additional soluble bicarbonates likely leached from the monitored topsoil). Amending the soils with wollastonite skarn also resulted in better green bean plant growth, as indicated by the fresh and dry biomass as well as the higher yields. WAS promoted robust plant growth, thus demonstrating its potential for use as a soil amendment.

### Study 2—Role of Silicon (Si) Released During Wollastonite Skarn Weathering on Plant Growth (Soybeans and Lettuce)

Soybean plant height, leaf area, and stem width were measured and are shown in Table 1. Plant height, leaf area, and stem width were all statistically significantly higher in soybean grown with wollastonite skarn (*p* < 0.05). After harvest, the number of bean pods per plant and the weight of each bean pod were measured to determine yield (per plant and total). The number of bean pods obtained from soybean grown with wollastonite skarn was statistically higher, and the mean mass of bean pods was similar in the two treatments, resulting in a 41% higher total yield. In summary, the wollastonite skarn amendment caused soybean to grow larger and faster and yield more beans.

**Table 1:** Effect of wollastonite skarn on soybean and lettuce growth.

**Soybean**	**Control**	**Wollastonite amended soil (WAS)**	***p*-value**
Plant height (cm)	59.10 ± 6.01	62.10 ± 6.43	0.2953
Leaf area (cm^2^)	47.43 ± 5.54	58.13 ± 9.29	0.0058
Stem width (cm)	5.80 ± 0.75	7.90 ± 1.70	0.0022
Number of bean pods per plant	12.20 ± 2.18	17.10 ± 4.50	0.0062
Mean mass (g) of bean pod	0.698 ± 0.20	0.704 ± 0.21	0.9486
Total mass (g) of bean pods (yield)	85.2	120.3	–
Silicon in leaf (wt.%)	0.34 ± 0.13	0.75 ± 0.14	0.0001
Silicon in stem (wt.%)	0.02 ± 0.01	0.04 ± 0.01	0.0003
Silicon in bean pods (wt.%)	0.04 ± 0.01	0.10 ± 0.02	0.0001
Silicon in root (wt.%)	BDL^*^	0.09 ± 0.05	0.0001
**Lettuce**	**Control**	**Wollastonite amended soil (WAS)**	***p*** **-value**
Plant height (cm)	37.00 ± 2.10	41.4 ± 1.96	0.0001
Leaf area (cm^2^)	167.44 ± 30.28	224.00 ± 18.00	0.0001
Leaf moisture (%)	92.68 ± 0.75	92.83 ± 0.90	0.6903
Silicon in leaf (wt.%)	BDL^*^	0.07 ± 0.02	0.0001

*Paired t-test (n = 10) yielded the p-values (p < 0.05 is significant)*.

* *Below AAS detection limit (0.01 wt.%)*.

Lettuce height and leaf area were measured, with wollastonite-amended lettuce growing both statistically significantly higher and larger. Results for the growth of lettuce are shown in Table 1. There was no statistically significant difference in moisture content, so the increase in size is attributable to increased biomass. The 34% higher leaf area in wollastonite-amended lettuce corresponds to a 34% greater yield. In summary, lettuce grown with wollastonite skarn grows larger and faster, which results in a greater yield.

Soybean silicon content in leaves, bean pods, stems, and roots was statistically significantly higher (using paired *t*-test, *p* < 0.05) when grown in WAS compared to the control, in all cases more than double. Lettuce was analyzed for silicon content and lettuce grown in WAS had detectable silicon content vs. undetectable content in those grown in the control treatment. These results show that the silicon that makes up a large part of the minerals wollastonite/diopside is bioavailable and readily taken up by both soybean and lettuce. Soybean leaves in particular accumulate a large amount of silicon (Ma and Yamaji, 2008). The increase in silicon may be a mechanism to explain the increased structural growth and yield characteristics observed in both soybean and lettuce. Studies have also shown that silicon plays an important role in aiding plants to better tolerate deficiencies in macronutrients and micronutrients (Miao et al., 2010; Pascual et al., 2016) and in protecting plants from diseases such as soybean rust (Lemes et al., 2011).

**Figure 3:**
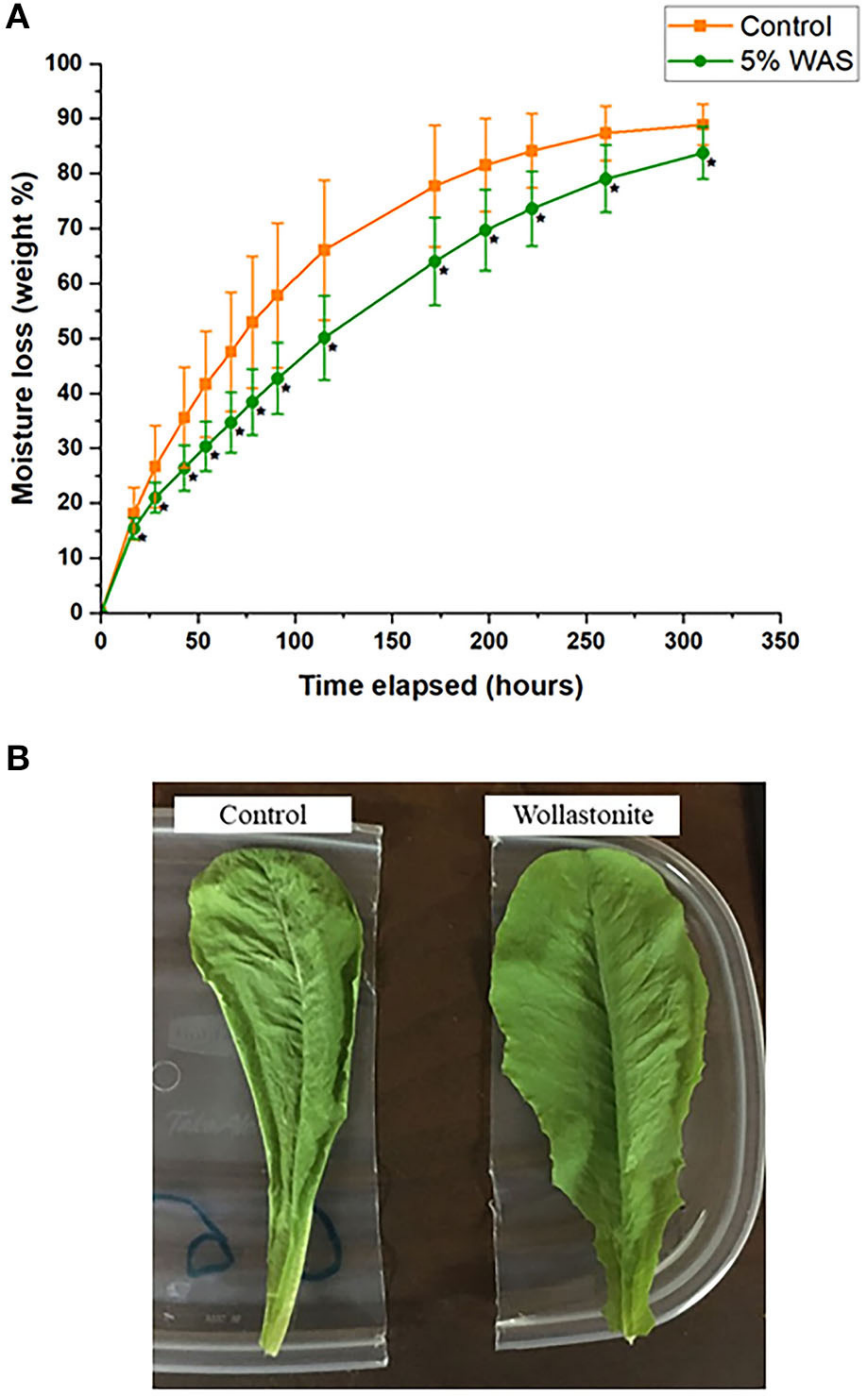
**(A)** Lettuce wilt experiment showing moisture loss over time for lettuce grown with and without wollastonite skarn (*points with statistical significance using paired *t*-test; *p* < 0.05), **(B)** Lettuce grown with and without wollastonite skarn shown after 115 h during the wilt test experiment.

Lettuce grown with and without wollastonite skarn were subjected to a wilt test experiment, the results of which are shown in Figure 3A. The wollastonite-amended lettuce had statistically significant lower moisture loss (using paired *t*-test, *p* < 0.05) throughout the test. The rate of moisture loss was roughly linear over the first 115 h, and if the rate of moisture loss is calculated linearly, then a rate of 0.34% moisture loss per day for WAS-grown lettuce is calculated. For control lettuce, a rate of 0.64% moisture loss per day is calculated, which is 88% greater than that for WAS-grown lettuce. Lettuce amended with wollastonite skarn was thus more resistant to wilting. During the wilt test experiment, it was also observed that subjective appearance changes associated with wilted lettuce were observed much faster in the control lettuce as compared to the lettuce grown in WAS. An example of this is shown in Figure 3B, where the lettuce grown in WAS appears much less wilted than the control lettuce after the same amount of time.

One mechanism of action could be the uptake of silica (SiO_2_) provided by the wollastonite/diopside weathering, which can create a more robust silicon cuticle layer on the leaf surface and limit water loss (Ma and Yamaji, 2008). Regarding silicon consumption for humans, based on available research studies, Si is not an essential nutrient for the human body, but an intake level of 20–50 mg per day is suitable and the safe upper limit was set at 700 mg/day for adults (Sadowska and Świderski, 2020). Considering the silicon content in the leaves of lettuce is shown in Table 1 and the typical amount of lettuce consumption per day (~75 g), the Si present in the lettuce grown with wollastonite skarn is not harmful for human consumption.

### Study 3—Effect of Co-application of Wollastonite Skarn and Urea (As Coated Fertilizer) on Plant Growth (Spring Rye), Carbon Sequestration, and Nitrogen Losses

#### Plant Biomass and N Content

**Figure 4:**
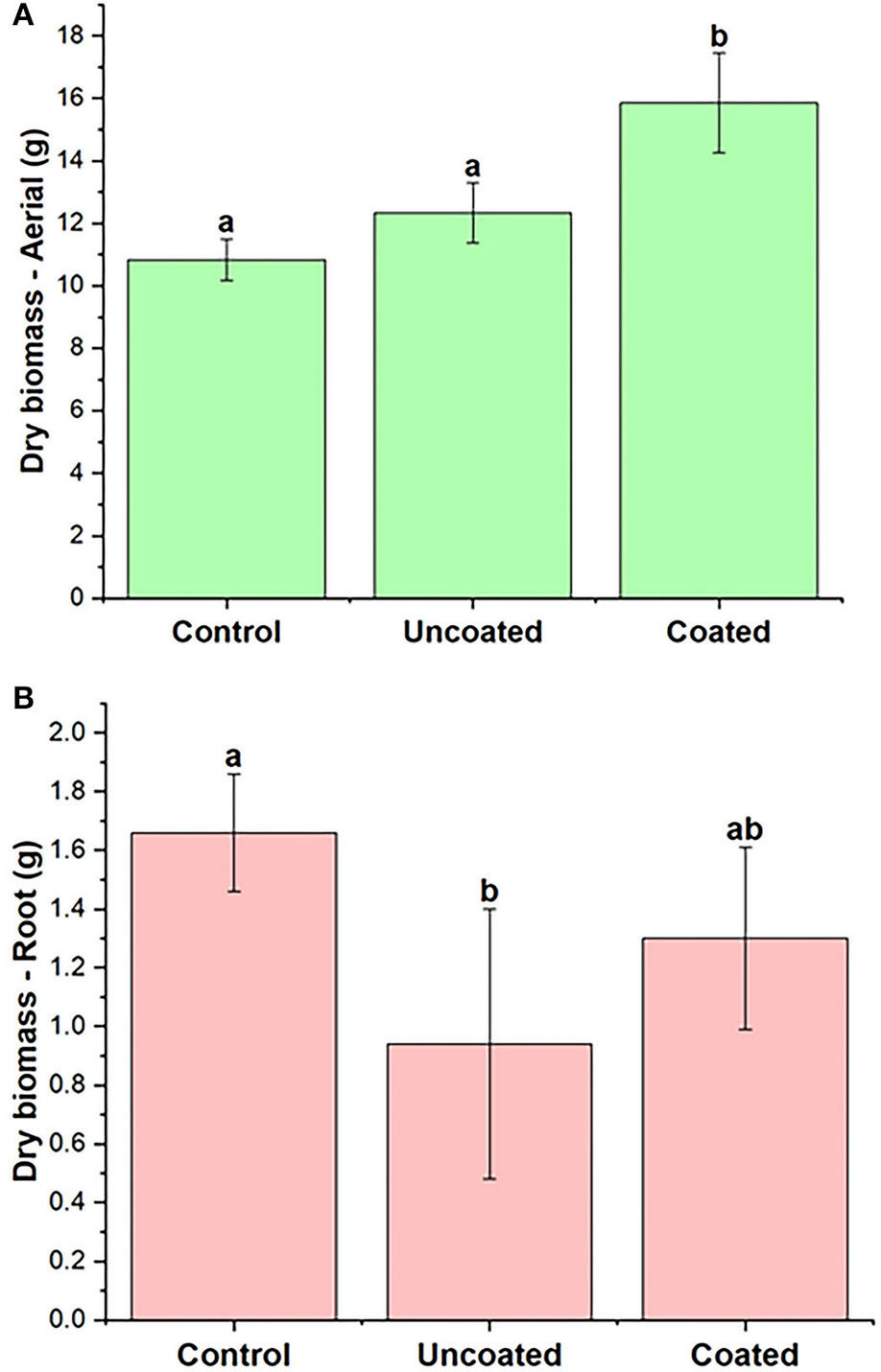
Spring rye **(A)** aerial **(B)** root dry biomass, representing control, uncoated, and coated treatments. Lowercase letters (a–e) indicate significant differences between treatments (*p* < 0.05).

Plant biomass was measured in terms of aerial and root biomass separately. The fresh aerial biomass in three treatments, control, uncoated, and coated, are significantly different from each other as shown in Table 2. The coated treatment had 66% higher fresh biomass than the control. Figure 4 shows the aerial dry biomass in control, uncoated, and coated treatments. Coated treatment had 46% higher aerial dry biomass than the control. The number of rye spikes was calculated after the harvesting. The number of rye spikes was in the order of Coated ≥ Control > Uncoated. All three treatments were statistically similar, and there was no apparent difference in the mean of all three treatments. The application of nitrogen to cover crops is beneficial to increase biomass production (Balkcom et al., 2018). Cover crops such as rye are known to scavenge mineral nitrogen and reduce nitrogen loss by leaching (Dabney et al., 2007; Kaspar et al., 2007; Lacey and Armstrong, 2015).

**Table 2:** Effect on three treatments of spring rye: control, uncoated, and coated.

**Treatment**	**Control**	**Uncoated**	**Coated**
Fresh biomass—Aerial (g/pot)	12.34 ± 0.82a	14.23 ± 1.10b	20.58 ± 0.72c
Number of rye spikes (per pot)	25.50 ± 2.38a	23.50 ± 2.08a	25.75 ± 2.22a
SIC (g/kg as CaCO_3_)	0.28 ± 0.15a	0.72 ± 0.05b	1.00 ± 0.09c
N in Plant—Aerial (mg/g)	18.55 ± 4.45a	19.17 ± 1.68a	15.74 ± 1.83a
N in Plant—Root (mg/g)	15.50 ± 2.34a	16.65 ± 1.80a	11.70 ± 3.35a
Soil pH	5.35 ± 0.05a	5.48 ± 0.03b	5.52 ± 0.04b
Salinity (mS/cm)	0.43 ± 0.05a	0.41 ± 0.04a	0.37 ± 0.04a
Soil Initial—Total Inorganic Nitrogen (mg/g)	0.229 ± 0.001a	0.265 ± 0.001a	0.265 ± 0.001a
Soil Final—Total Inorganic Nitrogen (mg/g)	0.120 ± 0.003a	0.122 ± 0.002a	0.124 ± 0.001a
Silicon—Aerial (wt.%)	0.14 ± 0.04a	0.20 ± 0.03a	0.19 ± 0.06a
Silicon—Root (wt.%)	0.19 ± 0.06a	0.30 ± 0.14a	0.20 ± 0.05a

*Data analyzed using Tukey's HSD test; lowercase letters (a–e) indicate significant differences (p < 0.05) between treatments*.

The nitrogen content present in aerial and root biomass was analyzed (Table 2), and it was found that aerial biomass contained more N than the root. N content in aerial and root parts of the uncoated treatment was higher than that for the control and the coated treatment, though differences were not statistically significant in terms of N content. However, differences are clearer by looking at N uptake (mg/pot). The N amount present in the control, uncoated, and coated treatments was calculated [dry biomass (g) × N in plant-aerial (mg/g)] to be 201.08 ± 2.94 mg, 236.37 ± 1.61 mg, and 249.48 ± 2.93 mg, respectively. Here, the coated treatment results in greater N uptake and thus greater fertilizer efficiency as defined by uptake vs. applicate rate (920 mg per pot).

#### Plant Height and Yield

**Figure 5:**
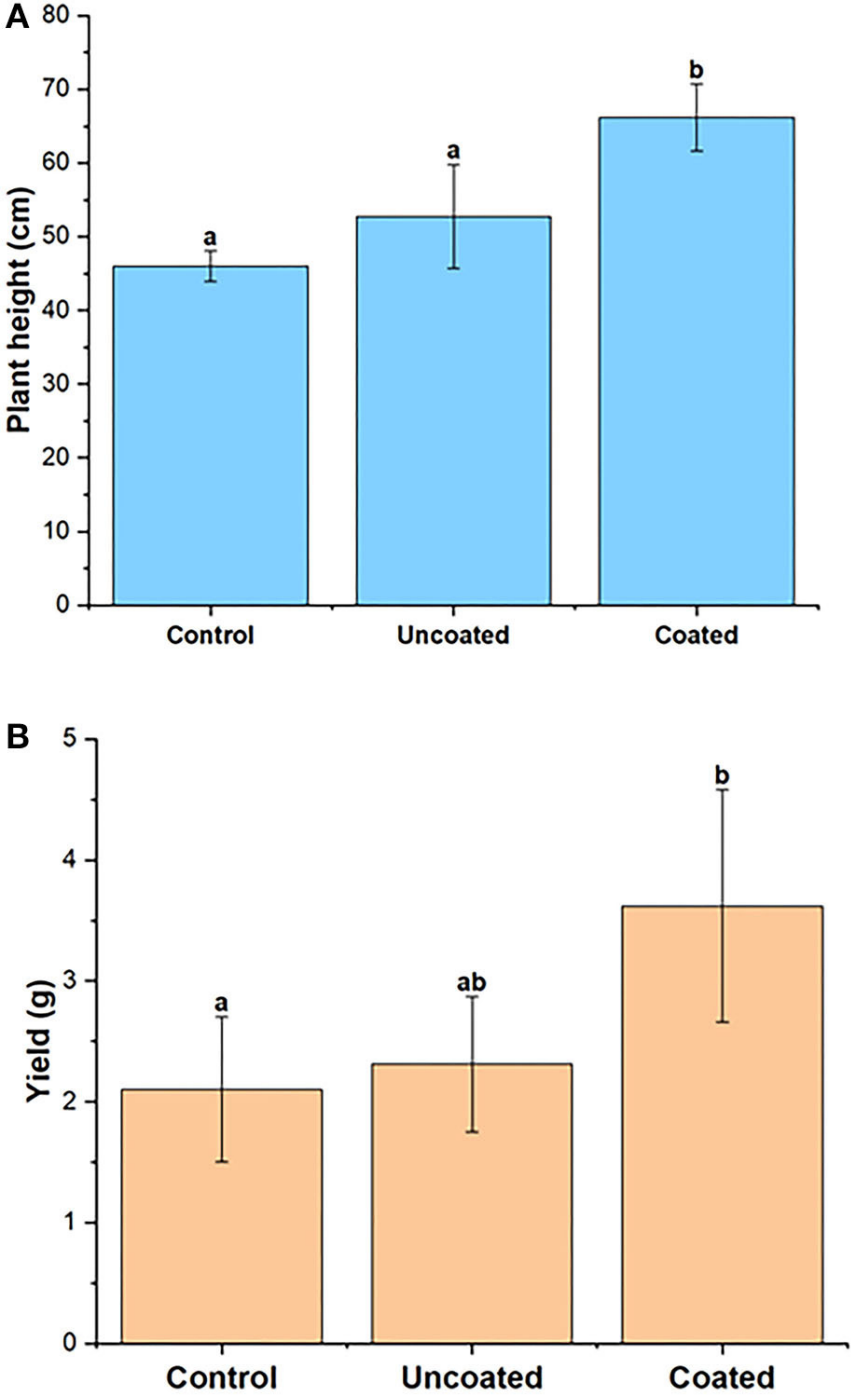
**(A)** Plant height and **(B)** yield of spring rye, representing control, uncoated, and coated treatments. Lowercase letters (a–e) indicate significant differences between treatments (*p* < 0.05).

The plant height of the spring rye was measured at the end of the experiment. The plant heights (in cm) of all the three treatments, control, uncoated, and coated, were 45.98 ± 2.08, 52.75 ± 7.04, and 66.18 ± 4.56, respectively. The statistical significance between treatments was significant, and coated treatment was statistically different from control and uncoated. Figure 5 shows that the highest height for the spring rye plants was observed in the coated treatment. The yield of each pot was calculated in terms of the total grain weight. The yield (in grams) was in order of coated (3.62 ± 0.96) > uncoated (2.31 ± 0.56) > control (2.1 ± 0.60). The mean in all three treatments was significantly different from each other. The highest yield was observed in coated treatment.

#### Silicon Uptake

**Figure 6:**
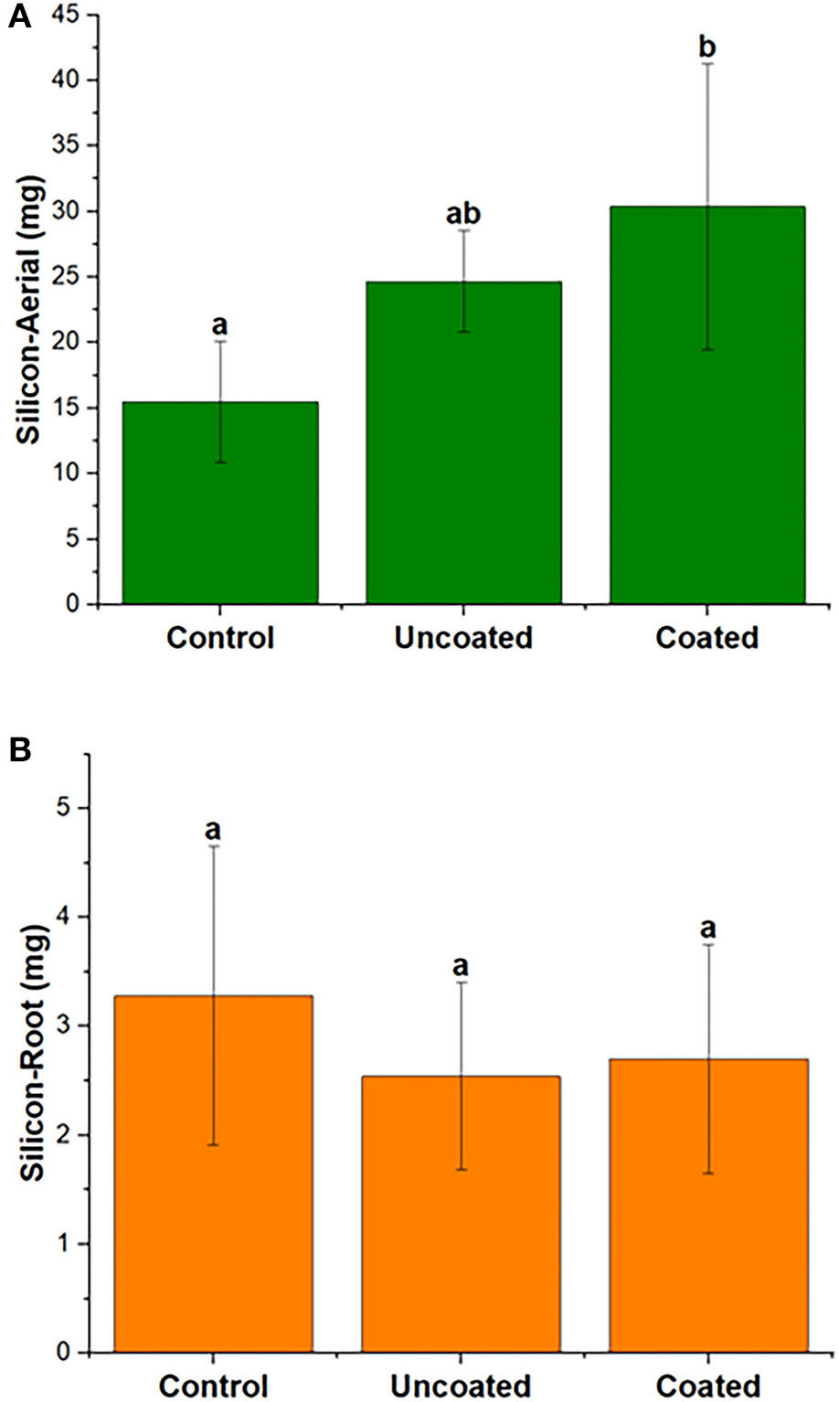
Total silicon of **(A)** aerial and **(B)** root biomass of spring rye (per plant), representing control, uncoated, and coated treatments. Lowercase letters (a–e) indicate significant differences between treatments (*p* < 0.05).

Silicon uptake in spring rye was investigated in aerial and root separately. Figure 6 shows silicon uptake (mg/pot) in aerial was higher than the root in all three treatments, and the highest uptake in aerial was observed in the coated treatment. The statistical significance for aerial and root in all treatments was insignificant, and the three treatments were significantly similar. According to Table 2, in the aerial part, when compared with the control treatment, silicon concentration was 43% higher in uncoated and 36% higher in coated treatments. In the root part, when compared with the control treatment, silicon uptake was 58% and 5% higher in the uncoated and coated treatments, respectively.

Silicon is the second most widely available element in the soil after oxygen. Silicon in the form of silicon dioxide (SiO_2_) contains 50–70% of the soil constituents. Rooting systems of all plants in soil are exposed to Si in their tissues, and the role of Si is overlooked in plant growth (Epstein, 1999; Ma and Takahashi, 2002; Richmond and Sussman, 2003). The plant root uptakes silicon from the soil in the form of Si(OH)_4_ (Silicic Acid) within a concentration range of 0.1–0.6 mM with a pH value below 9 (Ma and Takahashi, 2002). Various plants uptake the silicon based on their accumulating ability, and the concentration of Si ranges from 0.1 to 10% of plant dry weight (Epstein, 1999; Ma and Takahashi, 2002). The uptake of the Si mechanism in various plant species differs from each other. Mitani and Jian studied rice, cucumber, and tomato for silicon uptake, and they found that the accumulation of Si in these species was high, medium, and low, respectively (Mitani and Jian, 2005). Very few articles are available on silicon uptake in rye experiments and dynamics of uptake with fertilization effect. In a study by Jones and Handreck, they found that silicon accumulation in plants can be categorized into three groups (Jones and Handreck, 1967): highest values of silicon in the range of 10–15% on a dry weight basis found in wetland Gramineae (rice); the intermediate level at 1–3% found in rye and oats; and dicots having <1%. In the present study (Table 2), silicon uptake in spring rye in the context of% of dry weight basis in three treatments, control, uncoated, and coated, were 0.14, 0.20, and 0.19 and 0.19, 0.30, and 0.20 in aerial and root, respectively. In the present study, the soil pH was maintained between 5.3 and 5.7 for spring rye, which does not affect silicon uptake in all three treatments. All treatments have significant similarities in terms of soil pH. Jones and Handreck mentioned that silicon uptake in the plant depends on soil pH, species, transpiration, and nutrient supply. They found that the effect of soil pH varies from plant to plant, and other substances present in the soil, such as organic acids, organic matter, presence of iron and aluminum, also affect the silicon uptake (Jones and Handreck, 1967).

**Figure 7:**
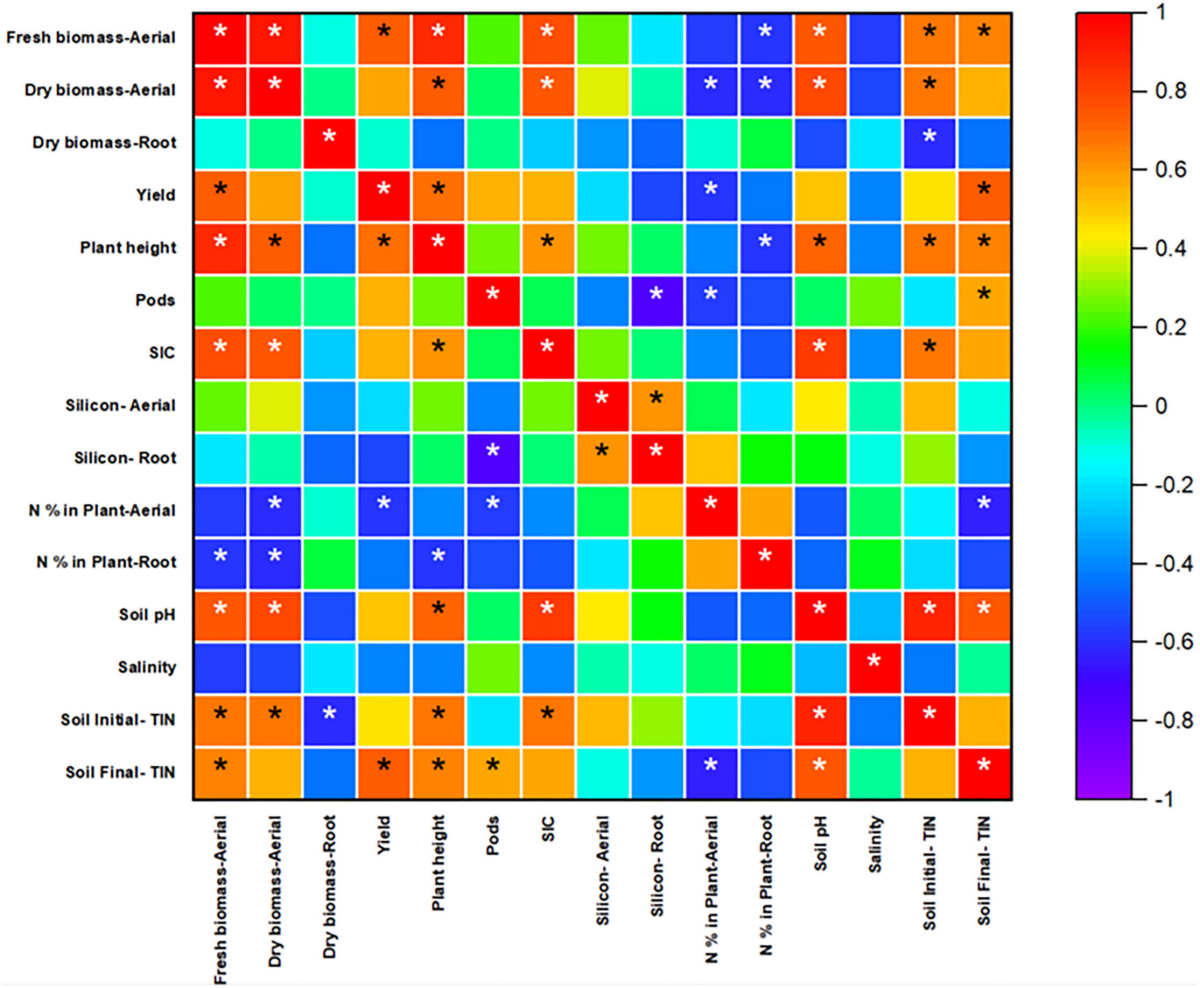
Pearson's correlation analysis for spring rye. The scale indicates the level of significance (−1 to 1) and stars indicate correlations that meet the significant level (*p* < 0.05).

The uptake capacity of plants in aerial and root parts depends on the plant metabolic process and the ability to determine silica concentration from the soil. The transpiration rate also affects the silica accumulation in plants. The concentration of silica increased with decreasing the transpiration rate in the plant system (Jones and Handreck, 1967). Wu et al. (2017) studied the effects of nitrogen fertilizer on rice and found that nitrogen fertilizer application led to a decrease in Si accumulation in rice biomass. With increasing nitrogen concentration, plant dry biomass weight increased, but it led to a reduction in Si uptake due to a decrease in the expression of OsLsi1 and OsLsi2 transporters, which play a significant role in Si accumulation in rice (Wu et al., 2017). In the present study, silicon was accumulated in roots compared to aerial parts. In spring rye, when control treatment was compared with the uncoated and coated treatments, silicon in aerial and root parts had a negligible difference. It showed that fertilizer application did not affect silicon uptake in aerial and root. Moreover, Pearson's correlation analysis (Figure 7) for Spring also showed that nitrogen present in plant biomass and soil is negatively correlated with silicon (aerial and root both), which was a correct indication from data analysis and also supported the references cited above.

#### Results Based on Experimental Analysis of Spring Rye Pot Soil

The soil parameters such as SIC (Soil Inorganic Carbon), pH, salinity, soil initial, and final inorganic nitrogen were analyzed (Table 2). The SIC content (in g/kg as CaCO_3_) was in the order of coated > uncoated > control. The initial soil pH was 5.47 ± 0.042 and recorded before starting the experiment. At the end of the experiment, the final soil pH for control, uncoated, and coated was 5.35 ± 0.05, 5.48 ± 0.03, and 5.52 ± 0.04, respectively. Soil pH for the uncoated and coated treatments was significantly different from the control. Salinity was measured, and there was not much difference in all three treatments. The soil's initial–total inorganic nitrogen (in mg/g) in the control, uncoated, and coated treatments was 0.23, 0.26, and 0.26, respectively. In the uncoated and coated treatments, the N application rate was similar, and application was made after the germination of seeds. The soil's final total inorganic nitrogen in the control, uncoated, and coated treatments was 0.120 ± 0.003, 0.122 ± 0.002, and 0.124 ± 0.0005 mg/g. All three treatments were statistically similar and represent the same level of the mean difference. Pearson's correlation test was conducted to see the correlation between the parameters and is presented in Figure 7.

In the spring rye study, soil pH in uncoated and coated was significantly different from control. The slight difference in soil pH could be a reason for the interaction of applied fertilizer and the presence of soil ions. Rye cover crops can grow in an optimum soil pH of 5.0–7.0 and tolerate the range of 4.5–8.0 (Grubinger, 2010). Soil pH governs the availability of plant nutrients and soil absorption capacity to retain them. Soil pH in the range of 5–8.5 favors cultivation (Gillman et al., 2002). Soil infertility is caused by extreme acidity (3–4) or alkalinity (8–10). In acidic soils, some ionic forms such as K^+^, Ca^2+^, Na^+^, Fe^2+^, Mg^2+^, ${\text{SO}}_{4}^{2-}$, and Cl^−^ dominate with aluminum (Al) with other organic ligands and OH (Lancashire et al., 1991). Soil systems are not confined or closed as they are always in a state of dynamic chemical equilibria and depend on input/outputs into the system, atmospheric condenzation, and gases entering or leaving the soil systems. Soil organic matter is aggregated on the topsoil profile, where soil pH buffering is governed by weakly dissociated organic acids. pH value below 5 leads to buffering in soil caused due to the presence of aluminosilicates (if they are present) and decomposition of clays. pH value in the neutral or moderately acidic range (5–7) is usually due to ion-exchange reaction associated with clays and organic matter. For pH value above 6.5, the concentration of carbonate and bicarbonate anions increases due to the presence of partly dissolved alkaline earth elements and alkaline (Haque et al., 2019). In a study by Guoju et al. (2012), they found that increasing winter temperature can impact the soil pH. The increased temperature of 0.5–2.0°C during the winter season can raise soil pH by 0.42–0.67 compared to no temperature change. Spring rye setup was kept in the greenhouse, but from December to early June, the average greenhouse temperature was increased by 3°C and that could impact the soil pH (Guoju et al., 2012).

In spring rye, the initial total inorganic nitrogen (ammonium + nitrate) in all three treatments was significantly similar. The nitrogen application to uncoated and coated treatments was similar and showed no difference. For coated treatment, N in the plant (aerial) in mg/g was the lowest among other treatments, but dry biomass weight was the highest among control and uncoated treatments. Multiplying dry biomass (g) and N (mg/g) in aerial showed that total nitrogen present in plant biomass was higher in coated than control and uncoated treatments. The presence of high nitrogen in coated treatment could be due to the slow-release properties of fertilizer. Nitrogen is one of the most important macronutrients for plant development, and the cover cropping strategy is beneficial for soil N balance and reduces N loss by leaching or erosion (Singh et al., 2018).

In the present study, 3.2 g of coated fertilizer was applied in both rye experiments. Each gram of coated fertilizer contains 18.85% of milled wollastonite skarn, which equals 0.60 g of wollastonite skarn in coated treatment. By considering the mineral composition in wollastonite skarn, and assuming that calcium released by the weathering of wollastonite and diopside can result in pedogenic carbonate formation (as observed by Dudhaiya et al., 2019), each gram of wollastonite skarn can potentially sequester 0.206 g of CO_2_ as pedogenic CaCO_3_. In the present study, the SIC level in spring rye soil for three treatments showed a positive correlation between the SIC level and the application of wollastonite skarn. SIC in coated treatment was highest among all three treatments, which shows that the presence of wollastonite and diopside in coated treatment was responsible for higher SIC compared to control. The lowest value of SIC in control directly correlated with soil acidification (soil pH dropped from 5.47 to 5.35 over the course of the experiment), which can cause losses of SIC (Raza et al., 2021).

## Conclusion

It can concluded from the present study that the wollastonite skarn amendment is a good strategy for supporting climate-smart agriculture practices to support plants in their growth (+29–46% in rye dry biomass), improve yield (+41–72% yield increase in soybean and rye), and produce quality (47% lower moisture loss of refrigerated lettuce). The current study found that applying WAS can help to improve the plant available Si in soil, as evidenced by higher Si uptake observed in lettuce (+600% in leaf), soybean (+121% uptake in leaf), and spring rye (+23–96% in areal biomass), and would save the cost of applying Si supplement for plants while also helping in pedogenic carbonate accumulation. The accumulation of Si in plants can improve the water retention capacity and reduce the moisture loss, which can improve the shelf life of plants. When applying nitrogen fertilizer along with WAS, it can help in providing Si and N to plants which is a win-win situation for farmers. It was demonstrated that coating the mineral onto the fertilizer can be a strategy for both easing the application of minerals to soil (due to co-application of fertilizer and mineral) and for improving the plant uptake of the nutrient. The coated treatment outperformed both the uncoated treatment and unfertilized control in terms of biomass production and N uptake. The three complementary studies undertaken here also point to the flexibility of applying wollastonite skarn in combination with fertilizers to different crops, different growth media (field soil, garden soil, and peat moss), and different settings (external microplot and greenhouse). A key to this flexibility is the synergism that wollastonite skarn continues to demonstrate, and this opens the door to further investigations but also encourages greater commercial adoption.

Still, there is a big research gap on how enhanced weathering minerals can change the metabolic process and transpiration rate in plants. Researchers should start focusing on the impact of silicon uptake through enhanced weathering of minerals on molecular mechanisms using genomic approaches, which can provide new insights into the mechanism of specific plant behavior. Silicon uptake in plants can also help in the biofortification of food (D'Imperio et al., 2016). Biofortification of food crops is a new concept and silicon-enhanced crops can help in silicon deficiency in human bodies, but limited evidence is available as of now.

## Data Availability Statement

The raw data supporting the conclusions of this article will be made available by the authors, without undue reservation.

## Author Contributions

Conceptualization: HJ, FH, SV, RS, and YC. Methodology, data curation, and writing—original draft preparation: HJ, FH, and SV. Writing—review and editing, supervision, and funding acquisition: RS and YC. All authors listed have contributed to this study and have approved this submission.

## Funding

We thank the Ontario Ministry of Agriculture, Food and Rural Affairs (OMAFRA) for providing a Highly Qualified Personnel (HQP) scholarship (UG-HQP-2020-100515) to HJ. We thank the financial support provided by the Ontario Agri-Food Innovation Alliance (Gryphon's LAAIR Product Development grant UG-GLPD-2021-101200) and the Canada First Research Excellence Fund (Food from Thought Product Development grant 499149).

## Conflict of Interest

The authors declare that the research was conducted in the absence of any commercial or financial relationships that could be construed as a potential conflict of interest.

## Publisher's Note

All claims expressed in this article are solely those of the authors and do not necessarily represent those of their affiliated organizations, or those of the publisher, the editors and the reviewers. Any product that may be evaluated in this article, or claim that may be made by its manufacturer, is not guaranteed or endorsed by the publisher.
